# Among‐tree variability and feedback effects result in different growth responses to climate change at the upper treeline in the Swiss Alps

**DOI:** 10.1002/ece3.3290

**Published:** 2017-08-30

**Authors:** Matthias Jochner, Harald Bugmann, Magdalena Nötzli, Christof Bigler

**Affiliations:** ^1^ Forest Ecology Department of Environmental Systems Science Institute of Terrestrial Ecosystems ETH Zurich Zurich Switzerland

**Keywords:** among‐tree variability, climate change, climate change feedback, climate–growth relationships, growth increase, growth trends, treeline

## Abstract

Upper treeline ecotones are important life form boundaries and particularly sensitive to a warming climate. Changes in growth conditions at these ecotones have wide‐ranging implications for the provision of ecosystem services in densely populated mountain regions like the European Alps. We quantify climate effects on short‐ and long‐term tree growth responses, focusing on among‐tree variability and potential feedback effects. Although among‐tree variability is thought to be substantial, it has not been considered systematically yet in studies on growth–climate relationships. We compiled tree‐ring data including almost 600 trees of major treeline species (*Larix decidua*,* Picea abies*,* Pinus cembra*, and *Pinus mugo*) from three climate regions of the Swiss Alps. We further acquired tree size distribution data using unmanned aerial vehicles. To account for among‐tree variability, we employed information‐theoretic model selections based on linear mixed‐effects models (LMMs) with flexible choice of monthly temperature effects on growth. We isolated long‐term trends in ring‐width indices (RWI) in interaction with elevation. The LMMs revealed substantial amounts of previously unquantified among‐tree variability, indicating different strategies of single trees regarding when and to what extent to invest assimilates into growth. Furthermore, the LMMs indicated strongly positive temperature effects on growth during short summer periods across all species, and significant contributions of fall (*L. decidua*) and current year's spring (*L. decidua*,* P. abies*). In the longer term, all species showed consistently positive RWI trends at highest elevations, but different patterns with decreasing elevation. *L. decidua* exhibited even negative RWI trends compared to the highest treeline sites, whereas *P. abies*,* P. cembra*, and *P. mugo* showed steeper or flatter trends with decreasing elevation. This does not only reflect effects of ameliorated climate conditions on tree growth over time, but also reveals first signs of long‐suspected negative and positive feedback of climate change on stand dynamics at treeline.

## 1 INTRODUCTION

Investigating plant population dynamics at their distribution limits is a key approach for understanding global change impacts (Luckman, 1996; Motta & Nola, 2001). High‐elevation treelines are one of the most prominent ecosystem boundaries (Körner, 2012; Tranquillini, 1979) and have attracted scientific interest for a long time (e.g., Brockmann‐Jerosch, 1919; Humboldt & Bonpland, 1807). Temperature is widely recognized as the major large‐scale driver determining upper treeline position around the globe (Brockmann‐Jerosch, 1919; Holtmeier & Broll, 2005; Humboldt & Bonpland, 1807; Körner, 2012) although some of the detailed processes are still subject to debate (e.g., Takahashi & Furuhata, 2016). Thus, the ongoing increase in global temperature (IPCC, 2013) affects the main limiting factor of tree growth at high elevations and may imply substantial alterations of regional and local forest dynamics.

Global land temperatures are changing, with a global mean increase of 0.85°C between 1880 and 2012 (IPCC, 2013). In mountain regions such as Switzerland, this trend is even more pronounced with a mean temperature increase of 0.35°C per decade over the last 30 years (Pepin et al., 2015; Rebetez & Reinhard, 2008). As temperature is the most important driver of treelines, global warming implies changes in treeline elevation and dynamics (Holtmeier, 2009; Körner, 2012; Tranquillini, 1979). Numerous effects of climate warming on upper treelines have been observed such as changes in tree physiognomy (Gehrig‐Fasel, Guisan, & Zimmermann, 2007; Holtmeier & Broll, 2005), changes in establishment rates (Holtmeier & Broll, 2011), an elevational advance of the ecotone (Gehrig‐Fasel et al., 2007; Harsch, Hulme, McGlone, & Duncan, 2009; Leonelli, Pelfini, Di Cella, & Garavaglia, 2011), an increase in stand density (Camarero & Gutiérrez, 2004; Gehrig‐Fasel et al., 2007), and increasing growth rates (Graumlich, 1991; Kipfmueller & Salzer, 2010; Motta & Nola, 2001; Salzer, Hughes, Bunn, & Kipfmueller, 2009).

A prominent consequence of global warming is changing growth rates of trees, which are determined by short‐ to long‐term climate effects on tree growth. Relationships between climate variables and secondary growth rates on the level of tree populations have been studied for a long time (e.g., Fritts, 1976). The effects of monthly or seasonal climate variables on population mean growth at the elevational or latitudinal treeline are well tested (Graumlich, 1991; Kipfmueller & Salzer, 2010), also for the European Alps (Babst et al., 2013; Carrer, Anfodillo, Urbinati, & Carraro, 1998; Frank & Esper, 2005; Oberhuber et al., 2008). However, some recent studies suggest considerable amounts of variability of the individual trees' growth response, which are not identifiable with standard analyses based on growth data at the population level (c.f. Buras et al., 2016; Carrer, 2011; Graumlich, 1991; Vanoni, Bugmann, Nötzli, & Bigler, 2016). To date, this among‐tree variability has rarely been accounted for, and the magnitude of its effect on the explanatory power of climate–growth analyses is suspected to be large but remains unclear. Additionally, the timing of growth appears to be temporally fine‐scaled (Kirdyanov, Hughes, Vaganov, Schweingruber, & Silkin, 2003; Lenz, Hoch, & Körner, 2013), and thus temporally higher resolved climatic data than only seasonal means should be used.

While rising temperatures per se improve life conditions for trees at high elevations, there are aspects of climate change that may amplify or dampen temperature effects on forest dynamics and create feedback effects. Changes in precipitation and cloud cover can alter water availability (Lloyd & Fastie, 2002), pathogen infection rates (Barbeito, Brücker, Rixen, & Bebi, 2013), and incoming radiation (Grace, Berninger, & Nagy, 2002). Due to the inverse relationship between tree lifespan and growth rate (Bigler & Veblen, 2009; Di Filippo, Biondi, Maugeri, Schirone, & Piovesan, 2012), improved growing conditions may enhance forest turnover (Bugmann & Bigler, 2011; Stephenson & Van Mantgem, 2005). Finally, changing stand density modifies canopy cover, thereby affecting tree‐to‐tree competition and root zone temperatures, which feed back to tree growth rates (Barbeito, Dawes, Rixen, Senn, & Bebi, 2012; Malanson et al., 2011; Wang et al., 2016). The interactions of these processes and the potential feedback effects make it challenging to predict tree population dynamics at the treeline ecotone in a changing climate, which in turn are expected to also have consequences for trees at lower elevations (e.g., Holtmeier & Broll, 2005; Seidl, Schelhaas, Lindner, & Lexer, 2009). Yet, knowledge about the future development of tree population dynamics may be inferred from past dynamics at the treeline and their temporal variability with respect to changing climatic conditions.

A distinguished feature of many tree‐ring chronologies—not only in the Alps but worldwide—is accelerated growth, often starting around the year 1950 (Kipfmueller & Salzer, 2010). While early studies attributed this increase to CO_2_ fertilization (e.g., La Marche, Graybill, Fritts, & Rose, 1984; Nicolussi, Bortenschlager, & Körner, 1995), more recent work has questioned this hypothesis (Handa, Körner, & Hättenschwiler, 2006). Lately, positive trends in growth rates are mostly ascribed to a direct climatic cause, especially rising temperatures and the extending growing season (Graumlich, 1991; Kipfmueller & Salzer, 2010; Motta & Nola, 2001; Salzer et al., 2009). Although tree‐ring data are often available as long time series, which renders them an ideal measure for multi‐decadal to centennial growth trend analyses, isolating the low‐frequency variability caused by abiotic influencing factors remains challenging. This variability is often confounded with the trees' age‐related trend, and the choice of detrending method for disentangling abiotic influences and age effects can greatly bias the results (Peters, Groenendijk, Vlam, & Zuidema, 2015). Furthermore, the sampling design may induce biases to the data that can void results on growth trends (Bowman, Brienen, Gloor, Phillips, & Prior, 2013; Nehrbass‐Ahles et al., 2014). Thus, it is advisable to thoroughly design dendroecological sampling and carefully choose the detrending method when attempting to analyze the long‐term effects of increasing temperatures on growth rates of treeline trees and identify feedback effects.

In the European Alps, a large share of the treeline rise can be attributed to the cessation of human activities (e.g., Gehrig‐Fasel et al., 2007; Leonelli, Pelfini, & Di Cella, 2009). When assessing the impact of climate change on treeline forest dynamics, biases due to the confounding influence of this decrease in land use as well as other regional treeline modulators, such as geomorphological processes, wind and snow damages need to be considered. While the cessation of human land use and geomorphological processes mainly affect studies on the establishment of trees above the current tree limits (Gehrig‐Fasel et al., 2007; Leonelli et al., 2009), other factors such as wind and snow can also influence short‐ and long‐term tree growth variability. When we try to isolate the impacts of increasing temperatures on tree growth variability, it is therefore necessary to rule out growth‐determining factors other than temperature as much as possible.

We carefully selected climatic treeline sites in three major climate regions of the Swiss Alps that are as undisturbed by non‐climatic drivers (e.g., land use, geomorphology, etc.) as possible, which makes them suitable for the analysis of the effects of recent climate change. The goals of our study were to evaluate the elevational dynamics of these treeline stands using stand structure and age data, analyze short‐term temperature–growth relationships of major treeline tree species at the population and individual tree level and quantify longer‐term growth trends at the population and tree level. Specifically, we ask the following research questions: (i) Are the analyzed treeline sites limited by temperature and do they show signs of suspected upward movement? (ii) Which monthly temperature variables control the annual growth of trees at the treeline and how do their effects differ among climate regimes, species, and individual trees? (iii) Do long‐term growth rates reflect the recently increasing temperatures due to climate change, and can we identify feedback effects on growth rates?

## 2 MATERIALS AND METHODS

### 2.1 Study site selection

The shape of treelines can help to identify sites that are undisturbed by human activities, as it is reasonable to assume that diffuse treelines are limited by temperature during the growing season while abrupt treelines are forced by climatic controls on seedling mortality or frost‐ and wind‐induced growth limitations (Harsch & Bader, 2011; Malanson et al., 2011). Additionally, the recent cessation or extensification of human activities in many regions of the Swiss Alps renders it challenging to differentiate between changes in the functioning and dynamics of treeline ecosystems that are caused by recovery from human land use and those that arise from climate change (Gehrig‐Fasel et al., 2007; Leonelli et al., 2009). For these reasons, forest “outposts” at treeline (Körner, 2012) were investigated in this study.

The study sites were chosen using multiple criteria. The regionally highest patches of forest in Switzerland were identified using Swiss land use statistics GEOSTAT (Gehrig‐Fasel et al., 2007). Following the assumptions of Körner and Paulsen (2004), these regionally highest patches of forest are most likely limited by temperature and not by anthropogenic activities. The climatic limitation of these sites was checked by examining changes in treeline position between historic and current aerial images and evaluating detailed descriptions of high‐elevation treelines from the early 20th century (Brockmann‐Jerosch, 1919; Hess, 1923; Imhof, 1900). During preliminary field surveys, the form of these treelines was assessed according to the conceptual model by Harsch and Bader (2011) to confirm temperature as a potential key factor determining tree growth. Still, we cannot exclude that some of the ongoing ingrowth and densification of the forests at the identified treeline sites is due to anthropogenic influences or abiotic treeline modulators other than temperature.

**Figure 1:**
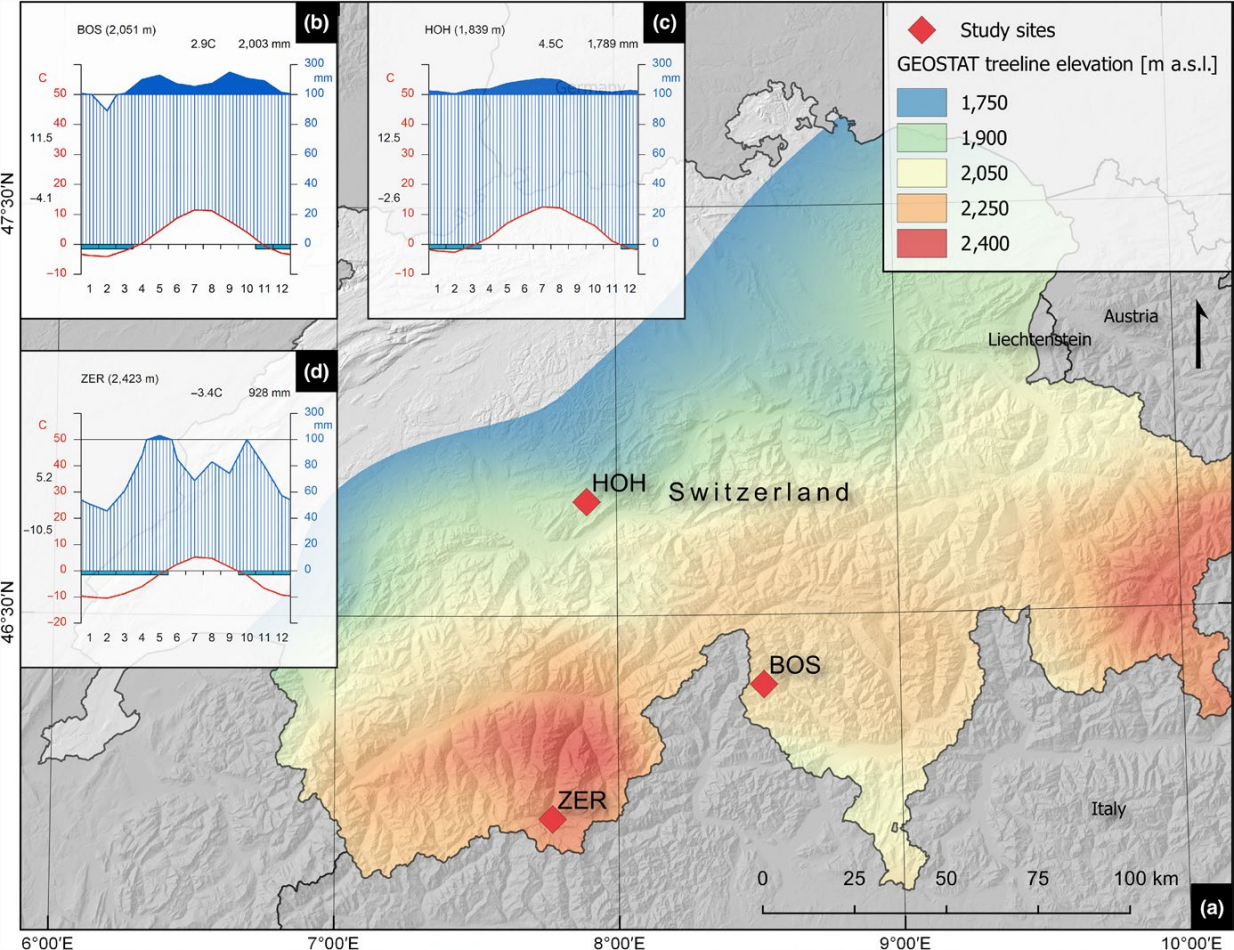
(a) Location of the three study sites Bosco/Gurin (BOS), Hohgant (HOH), and Zermatt (ZER) in Switzerland in the context of regional treeline elevation calculated using the Swiss land use statistics (GEOSTAT) and the method proposed by Gehrig‐Fasel et al. (2007). (b–d) Climate diagrams of the study sites employing gridded MeteoSwiss climate data using a 30‐year standard period from 1981 to 2010, showing distinct differences in precipitation sum and pattern between the Insubrian southern Alpine climate (b), the northern Alpine climate (c), and the central Alpine climate (d)

The following treeline sites in the Swiss Alps were selected, each containing multiple forest outposts: (i) Stavel Crastu near Bosco/Gurin (treeline at approximately 2,200 m a.s.l., for a photo see Fig. S1), (ii) the Hohgant massif near Schangnau (treeline elevation at approximately 2,000 m a.s.l., Fig. S2), and (iii) the Gugle ridge near Zermatt (treeline elevation at approximately 2,500 m a.s.l., Figures 1a and S3; Table 1). Each study area is located in a distinct climate region of the Swiss Alps: Bosco/Gurin is situated in the Insubrian southern Alps with high precipitation and high irradiation (Figure 1b), Hohgant in the northern Alps experiencing high precipitation and low irradiation (Figure 1c), and Zermatt in the central Alps with low precipitation and high irradiation (Figure 1d). These distinct differences in precipitation and radiation patterns enable us to qualitatively assess the impact of precipitation on the short‐ and long‐term growth variability, rather than using interpolated precipitation data, which would potentially introduce a high amount of uncertainty (Daly, 2006).

**Table 1:** Summary of the study area characteristics, dendroecological sampling, and remote sensing data

	Bosco/Gurin	Hohgant	Zermatt
Topography			
Slope range (°)	6–70	0–50	0–70
Expositions	Southwest, south	South, west, east	North, northwest, northeast
Dendroecological sampling			
Sampled species	*Picea abies*,* Larix decidua*	*Picea abies*,* Pinus mugo* ssp. uncinata	*Pinus cembra*,* Larix decidua*
No. of sampled trees	60 (spruce), 95 (larch)	85 (spruce), 83 (mountain pine)	95 (larch), 84 (Swiss stone pine)
Elevation range of sampled trees (m a.s.l.)	1,939–2,297	1,708–2,021	2,250–2,540
Tree height range (m)	2.5–26.4	2.2–25.0	2.0–24.0
Tree diameter at breast height (DBH) range (cm)	5.1–88.0	5.2–92.0	5.0–134.0
Mean tree ages ± standard deviation			
Upper 25%	36 ± 15	104 ± 55	94 ± 28
Middle 50%	54 ± 36	99 ± 63	68 ± 31
Lower 25%	72 ± 55	100 ± 42	115 ± 81
Remote sensing data			
Elevation range of data (m a.s.l.)	1,658–2,140	1,698–1,975	2,129–2,492
Area of canopy height model (ha)	68.5	87.5	141.7
Number of detected trees with height >2.5 m	16,461	10,289	13,480

### 2.2 Sampling design

At each study site, we defined three forest outposts as sampling plots. In each plot, 64 trees equally distributed over the prevailing tree species and four classes of diameter at breast height (DBH; 5–10, >10–15, >15–20, >20 cm) were sampled with increment borers, employing a stratified sampling and resulting in a “pseudo‐population” design (Nehrbass‐Ahles et al., 2014).

The sampled tree species included Norway spruce (*Picea abies*) and European larch (*Larix decidua*) at Bosco/Gurin, Norway spruce and mountain pine (*Pinus mugo* subsp. *uncinata*) at Hohgant, and Swiss stone pine (*Pinus cembra*) and European larch at Zermatt. Trees in each DBH class covered the complete elevation range of the plot. Two cores were taken per tree, one at breast height (130 cm) and one as close as possible to the root collar–shoot boundary. During coring, we always attempted to hit the pith. The estimation of tree age and primary growth rate from the stem base to breast height is essential and only possible when the pith is included in the sample. Each tree's coordinates, tree height, crown length, DBH, elevation, aspect, and slope were measured. We further recorded whether the tree grew with a single or multiple stems. In the laboratory, the increment cores were mounted on wooden supports, sanded and the ring widths measured with a resolution of 0.01 mm on a measurement device (Lintab 5, Rinntech, Heidelberg, Germany) according to standard dendrochronological procedures (Fritts, 1976). The ring‐width series were crossdated visually and quantitatively using COFECHA (Holmes, 1983) to ensure correct assignment of calendar years to each ring (cf. Table S1 for summary statistics of the tree‐ring‐width series).

### 2.3 Climate data

For the analysis of climate–tree growth relationships, gridded HISTALP monthly mean surface temperature (spatial resolution 5 arc minutes) was used (Chimani, Matulla, Böhm, & Hofstätter, 2013). To ensure correct representation of treeline temperature conditions in the HISTALP data, a total of 72 (eight per plot) temperature loggers (iButton DS1922L, Maxim Integrated) were installed, which recorded hourly 2 m air temperature between May 2014 and November 2015. The temperature loggers were protected from radiation by self‐developed Gill screens. A direct evaluation of HISTALP temperatures against in‐situ measurements was not possible, since the gridded data were only available until 2008. Therefore, we calculated Pearson correlations (*r*) between treeline stand monthly mean temperatures and all available monthly mean temperature records of MeteoSwiss climate stations. Stations with the highest correlations and longest time series were selected (station Locarno/Monti for Bosco/Gurin, *r* = .98; station Grimsel Hospiz for Hohgant, *r* = .98; station Col du Grand St. Bernard for Zermatt, *r* = .99) and used to analogously evaluate the HISTALP data (grid cell Bosco/Gurin, *r* = .97; grid cell Hohgant, *r* = .95; grid cell Zermatt, *r* = .98). HISTALP monthly mean temperatures were used from 1850 onwards. We did not use precipitation data for the analysis as (i) temperature is assumed to be the major limiting factor at the study sites (Körner, 2012), and (ii) long‐term precipitation data are only available from climate stations far from the study sites, which cannot be used due the highly inhomogeneous distribution of precipitation in mountainous terrain (Daly, 2006), and (iii) the available water capacity of a tree is not only defined by precipitation, but would need to be assessed in combination with soil characteristics and slope (Barij, Stokes, Bogaard, & Van Beek, 2007).

Long‐term trends of monthly mean temperature at each study site were quantified using time series derived from the nearest grid point of the HISTALP monthly mean surface temperature dataset (Chimani et al., 2013). Locally weighted smoothing splines of monthly temperature anomalies with regard to the 30‐year mean from 1971 to 2000 were calculated.

### 2.4 Tree height and age distribution analysis

To capture stand structure, the height distribution of trees in each stand was surveyed by means of 3D surface model reconstructions. We used largely overlapping high‐resolution (<10 cm/px) aerial imagery obtained with an unmanned aerial vehicle. Canopy height models were derived and single trees with a height >2.5 m automatically delineated (Hyyppa, Kelle, Lehikoinen, & Inkinen, 2001; Zarco‐Tejada, Diaz‐Varela, Angileri, & Loudjani, 2014). For each stand, the trees were grouped in three elevation classes, using the 25th and 75th percentile as class breaks. Then, tree height distributions were plotted for each elevation class.

To assess tree age distributions of the remote sensing sample, tree heights were converted to tree age employing site‐specific linear regressions of tree age and tree height. These linear models were calculated using the dendroecological sample, which included measured tree ages and tree heights (for the age–height relationships see Fig. S4). Tree ages were estimated by dating the pith of the cores taken at the base of the trees. Tree ages were then converted to ingrowth times and plotted analogously to the height distributions in three elevation classes. The elevational extent and the size of the stands that were analyzed with remote sensing data were larger than and slightly different to the dendroecological sampling and ranged between 1,658 and 2,140 m a.s.l. and approx. 70 ha at Bosco/Gurin, 1,698–1,975 m a.s.l. and approx. 90 ha at Hohgant, and 2,129–2,492 m a.s.l. and 140 ha at Zermatt (Table 1).

### 2.5 Analysis of short‐term temperature–growth relationships

Ring‐width indices were calculated from the raw ring‐width series. The series were restricted to start in 1850 to match with the climate data; data going longer back in time were not considered. The series were detrended using a 32‐year spline to remove the low‐frequency components of the data and amplify interannual variability, resulting in annual ring‐width indices (RWI_*t*_^sp^). Most previous studies on climate–growth relationships have used correlations between climate variables and tree‐ring chronologies representing the whole population (e.g., Carrer et al., 1998; Oberhuber et al., 2008), which do not account for among‐tree variability. In our study, we used ring‐width indices derived from individual trees and as predictors standardized monthly mean temperature variables of the current and previous year of ring formation (Equation (1)) to fit linear mixed‐effects models (LMMs; Pinheiro & Bates, 2004) for each tree species and study site. Seasonal mean temperatures were avoided, as previous studies suggest that shorter time periods better account for growth variability (Kirdyanov et al., 2003; Lenz et al., 2013). The initial LMMs were calculated in the following form:

(1) $${\text{RWI}}_{t}^{\mathrm{sp}}=\beta_{0}+\sum_{i=1}^{k}\beta_{i}\times T_{t}^{M}+\sum_{j=1}^{l}\beta_{j}\times T_{t-1}^{m}+b_{0}+\varepsilon_{t}$$

with RWI_t_^sp^ being a spline‐detrended RWI in the year *t*, β_i_ being the coefficients of the fixed effects, *T*_*t*_^*M*^ being monthly mean temperatures from current January until current October, *T*_*t*−1_^m^ from previous August until previous December, *b*_0_ being a random intercept with $b_{0}∼N\left(0,\;\sigma_{b_{0}}^{2}\right)$ , and $\varepsilon_{t}$ being the residual error in year *t* with $\varepsilon_{t}∼N\left(0,\;\sigma^{2}\right)$. All monthly mean temperatures were centered and scaled. Temporal autocorrelation of ${\mathrm{RWI}}_{t}^{\mathrm{sp}}$ was accounted for by adding an autoregressive parameter φ of order 1 (Pinheiro & Bates, 2004) to the model (Equation (2)):

(2) $$\varepsilon_{t}=\varphi \times \varepsilon_{t-1}+\eta_{t}$$

where $\varepsilon_{t-1}$ is the residual error in the previous year, and $\eta_{t}$ is a noise term with zero mean.

The best‐fitting model was inferred from the initial full LMM (Equation (1)) using information‐theoretic model selection (Burnham & Anderson, 2002). A repeated evaluation of all possible 2^15^ model combinations that included all combinations of variables shown in the full model (Equation (1)) was conducted using the Bayesian Information Criterion (BIC; Aho, Derryberry, & Peterson, 2014) and maximum likelihood estimation (ML; Burnham & Anderson, 2002). A model with a lower BIC score indicates a better‐fitting model.

After identifying the best‐fitting model for each species and site, the LMM with the random intercept was extended by adding random effects for those variables, which were selected as fixed effects. This model form was then refitted using restricted maximum likelihood (REML) estimation. The assumptions of the final models were investigated to ensure the validity of the interpretation of model outputs. Model diagnostics included checks for linearity, absence of collinearity and heteroscedasticity, and normality of residuals. All calculations were conducted in the R statistics software (R Core Team, 2016), using the packages *dplR* (Bunn, 2008), *nlme* (Pinheiro & Bates, 2004) and *MuMIn* (Barton, 2016).

### 2.6 Detection of long‐term growth trends

Trends in tree growth are often affected by biases introduced by the sampling design (Bowman et al., 2013; Brienen, Gloor, & Zuidema, 2012; Nehrbass‐Ahles et al., 2014) and age trend removal (Peters et al., 2015). A review of past studies on tree growth trends (Bontemps & Esper, 2011; Melvin & Briffa, 2008; Peters et al., 2015) led us to favor regional curve standardization (RCS; Becker, 1989; Briffa et al., 1992) to account for the trees' age trend. For RCS, the cambial age of the tree at coring height is needed. For cores that did not hit the pith, a geometric method was used to estimate the number of missing years to the pith based on ring curvature and the average of the first five measurable ring widths (Duncan, 1989). Cores with more than 10 missing rings were excluded to restrict uncertainty introduced by this estimation.

Again, LMMs were employed to describe the growth increment per species and site as a function of time. Following the initial hypothesis of temperature being the single most important determinant of growth, elevation was added as a predictor for ring‐width indices, which is a proxy for the different long‐term mean temperatures experienced by the trees and further climatic and other environmental influences along the elevational gradient. Furthermore, the interaction of time and elevation was added to the model to cover potentially differing trends of growth on different elevation (temperature) levels. To check for other confounding variables, we further tested models with combinations of these variables and any other tree variables (height, DBH, crown length, aspect, and slope) in an information‐theoretic model selection approach. The model with the lowest Akaike Information Criterion (AIC) was achieved with only time, elevation, and their interaction predicting ring‐width indices for all species and sites. The AIC was used in this case because it was undesirable to restrict the model's size as the objective was to check for the explanatory power of the variables mentioned above. Therefore, the original trend model (Equation (3)) was used:

(3) $$\begin{array}{cc} \log({\text{RWI}}_{t}^{\mathrm{RCS}})=\; & \beta_{0}+\left(\beta_{1}+b_{1}\right)\times t+\left(\beta_{2}+b_{2}\right)\times \text{elev}+(\beta_{3}\\ & +b_{3})\times \left(t\times \text{elev}\right)+b_{0}+\varepsilon_{t} \end{array}$$

with ${\mathrm{RWI}}_{t}^{\mathrm{RCS}}$ being the regional curve standardized RWI in year *t*, β_0_ to β_3_ the coefficients of the fixed effects, elev the tree's elevation, *b*_0_ a random intercept with $b_{0}∼N\left(0,\;\sigma_{b_{0}}^{2}\right)$, *b*_1_ to *b*_3_ random slopes, and $\varepsilon_{t}$ the residual error in the year *t* with $\varepsilon_{t}∼N\left(0,\;\sigma^{2}\right)$ . Analogously to the climate–growth LMMs, an autoregressive parameter φ of order 1 (Pinheiro & Bates, 2004) was added to each trend model (Equation (3)). As for the temperature–growth LMMs, compliance with model assumptions was checked. For each site and species, several elevation levels were used for predicting ring‐width indices over time in interaction with the elevation of the trees. Elevations were chosen to be the 0th, 20th, 40th, 60th, 80th, and 100th percentile of the elevation range (Table 1) of the data underlying the LMMs.

## 3 RESULTS

### 3.1 Tree height and age distribution of the forest outposts

The age distribution of the dendroecological samples across the elevation gradients indicates Bosco/Gurin to have the youngest trees overall, whereas Hohgant and Zermatt have trees of similar age (Table 1). Bosco/Gurin yields a tree age of 36 ± 15 years (mean ± standard deviation) at the highest 25% and an increase to 72 ± 55 years when going down to the lower end of the sample. At Hohgant and Zermatt, the trees across the gradient are older, with tree ages at the upper 25% of 104 ± 55 years and 94 ± 28 years, respectively. At Hohgant, the mean ages are similar across the entire gradient, and at Zermatt there is a decrease in mean age in the middle 50% and again an increase in the lower 25% of the gradient.

**Figure 2:**
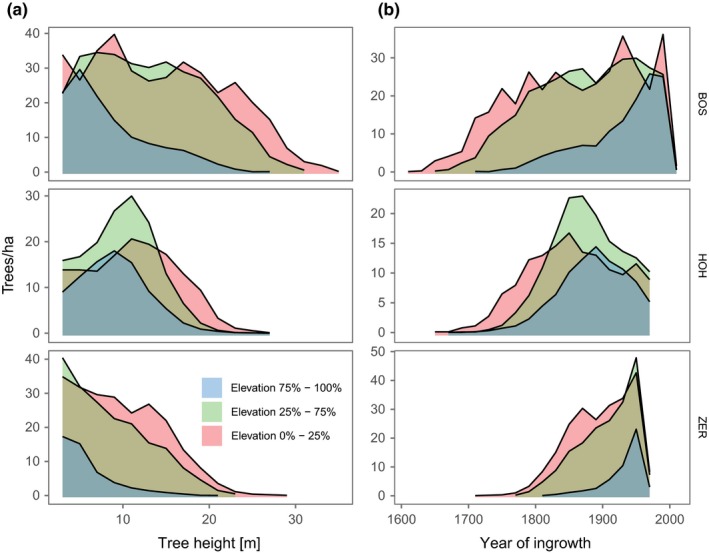
Tree height (a) and age (b) distribution at Bosco/Gurin (BOS), Hohgant (HOH), and Zermatt (ZER). Blue: upper 25% of trees in the sampled areas; green: central 50% of trees; red: lower 25% of trees. Tree ages were estimated using site‐specific linear regressions of the form Age_tree_ = *b*_0_ + *b*_1_ × height_tree_ (see also Fig. S4)

Average stand density across the entire elevational gradient was 277 trees/ha at Bosco/Gurin, 128 trees/ha at Hohgant, and 153 trees/ha at Zermatt; for the highest 25%, densities were 129, 89, and 48 trees/ha, respectively. At all study sites, the absolute number of trees in the highest 25% of the gradients was lower than within the lowest 25% or the central portion (25%–75%) of the elevation gradients (Figure 2). However, tree height distributions varied across study sites and elevation gradients. With increasing elevation, the distributions prominently shifted toward lower tree heights (Figure 2a).

The age distribution of the remotely sensed trees in the highest 25% of the elevation gradients approximated a negative J‐shaped curve of ingrowth time at Bosco/Gurin and Zermatt, while at Hohgant it was closer to a right‐skewed unimodal shape (Figure 2b). The lower parts of the stands at Hohgant also showed unimodal, bell‐shaped ingrowth time distributions. The distributions of the lower quantiles at Bosco/Gurin featured multiple modes and were right‐skewed, while at Zermatt they were close to negative J‐shaped curves.

Linear regressions of tree age and tree height in the tree‐ring dataset (Table 1) revealed highly significant (*p* values <10^−10^) positive relationships between age and height at all sites (Fig. S4). The strength of the relationship varied and was strongest at Bosco/Gurin ($R^{2}=0.48$ ), moderate at Hohgant ($R^{2}=0.43$ ), and lowest at Zermatt ($R^{2}=0.27$ ).

### 3.2 Analysis of short‐term temperature–growth relationships

**Figure 3:**
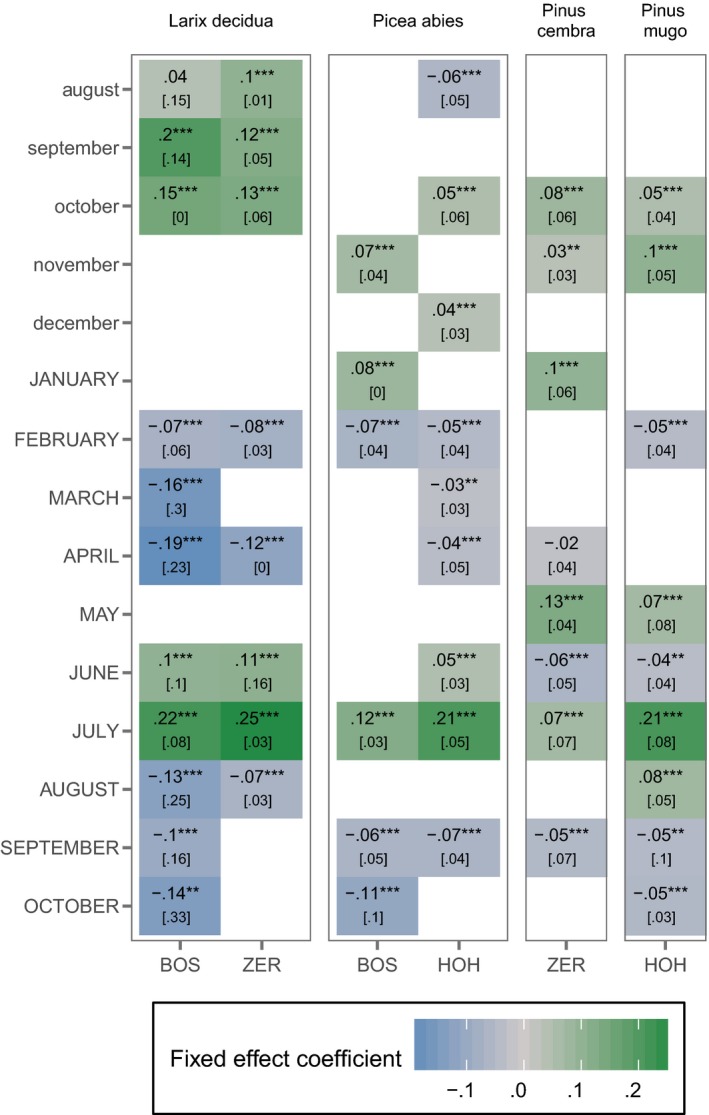
Temperature–growth relationships as calculated with LMMs (Equations (1) and (2)). Numbers and tile colors represent the fixed effect sizes of the best‐fitting temperature–growth models (lowest BIC) of *Larix decidua*,* Picea abies*,* Pinus cembra*, and *Pinus mugo* at all study sites. Significance levels of the fixed effects are indicated by * for *p* < .05, ** for *p* < .01, and *** for *p* < .001; for the absolute *p* values, see Fig. S5. The smaller numbers in brackets are the standard deviations of the random effects

The number of temperature variables affecting growth variability that were included in the LMMs showed considerable variability between species and sites (Figure 3), ranging from six monthly variables for spruce at Hohgant to eleven variables for larch at Bosco/Gurin. Also, the absolute values of the coefficients varied considerably, with larch having fixed effect values mostly >0.1, whereas spruce, mountain pine, and Swiss stone pine had values mostly <0.1 (Figure 3). Still, only two fixed effects were not significant (previous August for larch and current April for Swiss stone pine at Zermatt); all other coefficients featured *p* values <0.01 (Figure 3). Since our approach included multiple tests, the standard errors and *p* values were biased to some extent. Still, in most cases, the absolute *p* values lie at least one magnitude below the most rigorous significance level of *p* < 0.001 (Fig. S5). As the temperature variables were centered and scaled in the LMMs, a fixed effect of 0.1 indicates a 10% increase in ring‐width indices with a temperature increase by one standard deviation.

Larch showed a pattern of four clearly distinguishable seasonal periods at both Bosco/Gurin and Zermatt: positive fixed effects for previous year's late summer and fall temperatures (previous August, September, and October at both sites), negative fixed effects for current year's late winter and spring (February, March, and April at Bosco/Gurin, February and April at Zermatt), positive fixed effects for current year's mid‐summer (June and July at both Bosco/Gurin and Zermatt), and negative fixed effects for current year's late summer and fall (August, September, and October at Bosco/Gurin and August only at Zermatt). The highest positive values for both Bosco/Gurin (0.22) and Zermatt (0.25) resulted for current year's July, and the lowest negative values (−0.19 and −0.12, respectively) were found for current year's April (Figure 3).

Similar to larch, spruce featured significant seasonal periods for current year's late winter and spring, mid‐ and late‐summer as well as fall at both Bosco/Gurin and Hohgant (Figure 3). However, there were no clear seasonal effects for previous year's fall. The majority of absolute effect values for spruce at both sites was considerably lower, mostly below 0.1. As for larch, the highest positive fixed effects were found for July with 0.12 at Bosco/Gurin and 0.21 at Hohgant (Figure 3).

For Swiss stone pine and mountain pine, there were no clear seasonal patterns (Figure 3). While for mountain pine, current year's July temperature effect was notably higher (0.21) than the other fixed effects, the Swiss stone pine model featured generally low absolute values of coefficients with the highest positive coefficient in current year's May.

With the inclusion of random intercepts and slopes in the LMMs, among‐tree variability may be quantified in relation to the fixed effects. The standard deviations of the random effects showed considerable variability due to individual tree characteristics and tree‐specific environmental conditions (Figure 3). Similar to the fixed effects, larch exhibited the highest among‐tree variability. However, for current year's July (highest positive fixed effect), the standard deviation of the random effect was notably low, that is, tree growth responded in a similar way to temperature. This is also true for spruce and mountain pine, showing standard deviations of a comparable magnitude as the fixed effects in most months, except for July. Swiss stone pine, with its differing behavior featuring May as the month with the highest positive fixed effect, also has the relatively lowest standard deviation of the random effect in May.

Model diagnostics showed compliance of all temperature–growth LMMs with the assumptions of linearity, absence of collinearity and heteroscedasticity, as well as independence of the predictor variables. Still, the residuals partly exhibited a heavy‐tailed distribution, which was due to a small number of trees in each model causing large residuals, mostly toward the right end of the distribution.

### 3.3 Detection of long‐term growth trends

**Figure 4:**
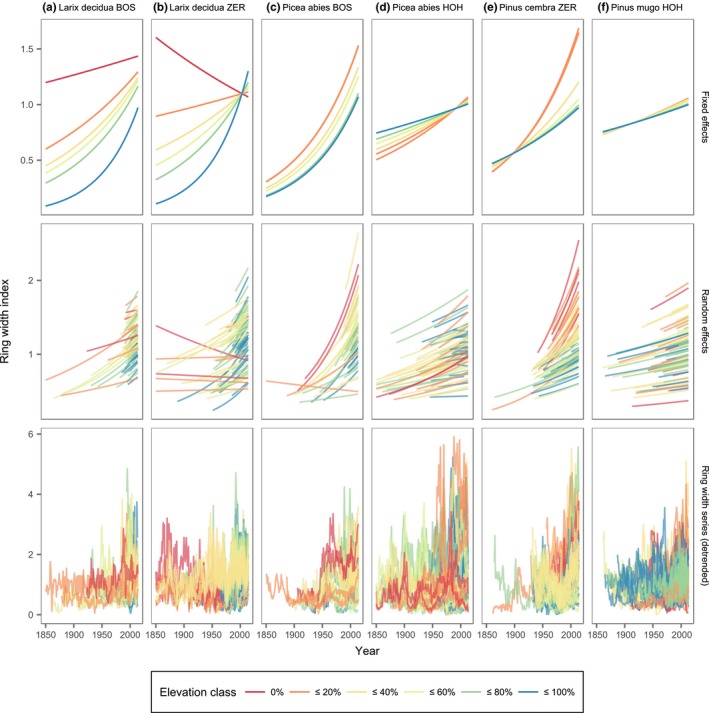
Ring‐width indices over time of the four tree species at the study sites Bosco/Gurin (BOS), Hohgant (HOH), and Zermatt (ZER) (a–f). The first row shows predictions based on the fixed effects coefficients at five different levels of elevation (0%, 20%, 40%, 60%, 80%, and 100% of the elevation range covered by the sampled trees at each study site, cf. Table 1). The second row shows predictions based on the random effects for each tree included in the model. The third row contains the original detrended ring‐width series that the models are based on. Both the predictions using the random effects and the ring‐width series are colored according to the elevation class of the individual trees

The predictions of tree‐ring‐width index (${\mathrm{RWI}}_{t}^{\mathrm{RCS}})$ using LMMs (cf. Equation (3), Table 2) revealed a positive growth trend across all species, sites and elevations although its strength varied considerably (Table 2; Figure 4). For larch, a strong positive trend at the highest elevations at both Bosco/Gurin and Zermatt was evident, but declined with decreasing elevation at both sites (Table 2; Figure 4a,b). There was a weak negative trend for predicted ring‐width index (${\mathrm{RWI}}_{t}^{\mathrm{RCS}}$ ) at the lower end of the elevation range at Zermatt. Note that the elevation range of the sampled trees and therefore also the range of elevations for the predictions was twice as large at Zermatt compared to Bosco/Gurin. For larch, time, elevation, and their interaction were significant at the levels of *p* < .05 (Bosco/Gurin) and *p* < .001 (Zermatt), respectively. Among‐tree variability was generally low for larch as indicated by the standard deviation of the random effects relative to the fixed effects (Table 2; Figure 4a,b).

**Table 2:** Summary of the model parameters of the LMMs for growth trend analysis for European larch (*L. decidua*), Norway spruce (*P. abies*), Swiss stone pine (*P. cembra*), and mountain pine (*P. mugo*) (cf. Equation (3))

Species	sampling site	Fixed effects (estimate ±*SE* [*p* value])				Random effects (*SD*)				Autoregressive parameter
		β_0_	β_1_	β_2_	β_3_	*b*_0_	*b*_1_	*b*_2_	*b*_3_	φ
*L. decidua*	Bosco/Gurin	259 ± 106 [0.015]*	−0.127 ± 0.053 [0.017]*	−0.126 ± 0.050 [0.013]*	6 × 10^−5^ ± 2 × 10^−5^ [0.013]*	0.184	4 × 10^−5^	8 × 10^−5^	1 × 10^−8^	0.63
	Zermatt	281 ± 48 [<0.001]***	−0.140 ± 0.024 [<0.001]***	−0.123 ± 0.020 [<0.001]***	6 × 10^−5^ ± 1 × 10^−5^ [<0.001]***	0.084	1.4 × 10^−4^	2 × 10^−5^	3 × 10^−8^	0.69
*P. abies*	Bosco/Gurin	27 ± 132 [0.84]	−0.010 ± 0.066 [0.88]	−0.022 ± 0.062 [0.72]	1 × 10^−5^ ± 3 × 10^−5^ [0.76]	2.245	2.3 × 10^−3^	4.03 × 10^−3^	1.51 × 10^−6^	0.83
	Hohgant	−250 ± 231 [0.28]	0.126 ± 0.117 [0.28]	0.126 ± 0.119 [0.29]	−6 × 10^−5^ ± 6 × 10^−5^ [0.29]	0.224	6 × 10^−5^	1.5 × 10^−4^	3 × 10^−5^	0.84
*P. cembra*	Zermatt	−203 ± 128 [0.11]	0.107 ± 0.064 [0.09]	0.077 ± 0.052 [0.14]	−4 × 10^−5^ ± 3 × 10^−5^ [0.12]	0.017	6 × 10^−5^	1 × 10^−5^	5 × 10^−8^	0.87
*P. mugo*	Hohgant	−42 ± 114 [0.71]	0.022 ± 0.058 [0.71]	0.019 ± 0.059 [0.74]	−1 × 10^−5^ ± 3 × 10^−5^ [0.73]	0.330	1 × 10^−4^	2 × 10^−5^	0	0.75

The coefficients relate to the intercept (β_0_) and the slopes of the time (β_1_), the elevation (β_2_), and the interaction of time and elevation (β_3_). *SE* refers to the standard error, *SD* to the standard deviation. Significance levels of the fixed effects are indicated by * for *p* < .05, ** for *p* < .01, and *** for *p* < .001.

For spruce at Bosco/Gurin, the LMM revealed weaker effects of time and elevation, featuring a stable positive growth trend on all elevation levels and decreasing absolute ring‐width indices with elevation (Figure 4c), but the standard deviation of the random effect for the intercept was high relative to the fixed effect intercept (Table 2). Conversely, the LMM for spruce at Hohgant showed weaker effects of time and elevation but a stronger interaction, resulting in a strengthening of the positive trend with decreasing elevation (Figure 4d). Both spruce LMMs did not indicate any highly significant effects.

For Swiss stone pine at Zermatt, the LMM yielded overall increasing ring‐width indices over time and a decreasing intercept of ring‐width indices with elevation (Figure 4e). The interaction term caused a strengthening of the positive growth trend with decreasing elevation. *p* values for the fixed effects were nearly significant (Table 2). Among‐tree variability for all effects was low.

For mountain pine at Hohgant, the low fixed effects of time and elevation (Table 2) caused a rather weak positive trend of ring‐width indices and decreasing base level of ring‐width indices with increasing elevation (Figure 4f). There was a constant positive growth trend across elevations. However, *p* values indicated that the trend was not significant.

### 3.4 Monthly mean temperature anomalies over time

**Figure 5:**
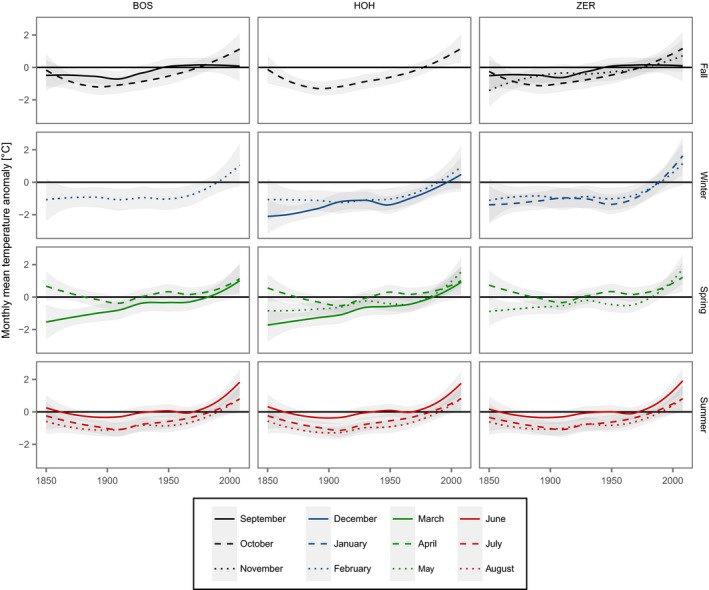
Locally weighted smoothing of monthly temperature anomalies with regard to the 30‐year mean from 1971 to 2000 (due to the length of the used HISTALP data, the new WMO standard reference period 1981–2010 could not be used). For each site (Bosco/Gurin: BOS; Hohgant: HOH; Zermatt: ZER), only the months that were included in the temperature–growth LMMs (Figure 3) are displayed. The grey shading indicates the 95% confidence intervals. For the sake of clarity, the single months are displayed in seasonal facets (rows)

To assess whether growth trends were associated with trends in climate variables, monthly mean temperature anomalies relative to the 1971–2000 reference period were calculated for 1850–2008, showing increasing temperatures at all sites and in all months (Figure 5). Patterns and strength of the temperature trends varied considerably among individual months, but only little between sites. All months except September exhibited a clear increase in temperature, starting around 1950 at the latest. The strongest temperature increase for all sites was found for June, featuring relatively constant temperatures around the 1971–2000 June mean until shortly after 1950, followed by a pronounced rise of almost 2°C. The weakest warming was evident for September, with mean temperatures staying relatively constant over the entire time period. The absolute increase in September mean temperature amounted to approximately 0.5°C since 1850.

## 4 DISCUSSION

### 4.1 Tree height and age distribution of forest outposts: implications for population dynamics and migration

Stand size and age structure analyses can reveal developmental patterns and may therefore provide evidence whether the sampled stands are largely free of anthropogenic disturbance, and can inform about their successional phase (Veblen, 1986). In our study, stand densities were uniformly low, especially in the highest parts of the stands, and similar to undisturbed treeline sites in the Polar Ural Mountains and interior Alaska (Lloyd & Fastie, 2003; Mazepa, 2005). Both the distribution of tree heights and ingrowth times in the uppermost 25% of the elevation gradient at Bosco/Gurin and Zermatt feature uneven age distributions and reverse J‐shaped height distributions (McCarthy & Weetman, 2006; Shorohova, Kuuluvainen, Kangur, & Jogiste, 2009). Thus, we conclude that these stands are forest outposts that have established prior to the beginning of the current warming trend, and do not appear to be moving uphill yet (cf. Harsch et al., 2009). At Zermatt, this is additionally backed by the mean ages of the trees included in the sample, indicating the trees to have grown at the highest elevations also before the beginning of the pronounced temperature increase (cf. Figure 5). However, the dendroecological sample of Bosco/Gurin yields trees at the highest elevations that are younger on average, which may indicate more recent ingrowth and a beginning advance of this treeline.

At Hohgant, however, the stands at the highest elevations are rather even‐aged, but still have relatively more trees with recent ingrowth time than in the lower elevation quantiles. This relative abundance of young trees and the lack of older trees at the highest elevation quantiles may be caused either by a rising treeline or due to harsh environmental conditions that promote fast turnover rates caused by frequent dieback (Lloyd & Fastie, 2003). While the latter would provide evidence for a climatic (i.e., anthropogenically undisturbed) treeline (Harsch & Bader, 2011), the former cause could indicate a combined effect: treeline rise due to ameliorating growth conditions and the recent cessation of human activities. The age distribution of the dendroecological sample rather points to the latter, yielding a mean tree age exceeding the start of the pronounced temperature increase (cf. Figure 5). Also, in case of the cessation of, for example, pasturing, one would expect many young trees to establish, but also the presence of a few remaining large old trees, thus leading to a bimodal distribution. This was not evident for any of the highest stands at the three study sites.

### 4.2 Analysis of short‐term temperature–growth relationships

The quantification of among‐tree variability and an analytical zooming in, below the population level, has long been stressed to be important (Carrer, 2011; Graumlich, 1991; Vanoni et al., 2016), but has hitherto been neglected in the analysis of climate–growth relationships. In other studies, temperatures of many and quite different months were found to be related to population mean growth across tree species at treeline in the European Alps, even with large differences for single species. Still, there is a stronger tendency of larch growth to be determined by temperatures over longer periods in the current year as well as in the year preceding growth ring formation (King, Fonti, Nievergelt, Büntgen, & Frank, 2013) compared to other species at treeline in the Alps. Based on “classical” correlation analyses, growth of Swiss stone pine and mountain pine, for example, tends to be explained by mean temperatures of only 1 or 2 months or summer seasonal means (Carrer et al., 1998; Lenz et al., 2013; Oberhuber et al., 2008). These findings are partly supported by our results, showing larch at Bosco/Gurin to include the largest number of monthly temperature variables in the LMM (Figure 3). However, our use of an information‐theoretic model selection of LMMs resulted in a substantially higher number of significant covariates also for spruce, Swiss stone pine, and mountain pine. This can largely be ascribed to the capacity of LMMs to explain not only a mean population signal, but also to account for among‐tree variability in the estimation of relevant covariates by employing tree‐specific random effects.

The species‐ and site‐specific temperature–growth LMMs showed common patterns, but also slightly differing findings. The fixed effects exhibited clear seasonal periods for larch and spruce growth at both sites, but somewhat less clear patterns for Swiss stone pine and mountain pine (Figure 3). Still, all LMMs yielded a strong positive effect of temperature in June/July (larch, spruce) and May/July (Swiss stone pine, mountain pine), forming periods of strong positive effects of summer temperature regardless of tree species and site. This generally agrees with previous studies using correlation analyses (Carrer et al., 1998; Graumlich, 1991; King, Gugerli, Fonti, & Frank, 2013; Kirdyanov et al., 2003; Oberhuber et al., 2008). Growth of Swiss stone pine and mountain pine does not seem to be driven by temperatures during a continuous period of summer months, as for both species the positive effects of May and July were interrupted by weak negative effects in June. In their study on mountain pine, Lenz et al. (2013) explained this pattern with temperatures early in the growing season to determine the width of the cambium and therefore to be the main determinant of ring width. Temperatures later in the growing season were assumed to determine the earlywood–latewood ratio. Given the similar pattern in our study, this explanation may also apply to Swiss stone pine, but with a somewhat higher impact of cambium formation on ring width than the earlywood–latewood ratio. For all species and sites, the random effects of these months exhibited low standard deviations relative to the fixed effects, thus indicating low among‐tree variability and the high importance of summer temperatures for synchronizing tree growth at treeline, regardless of tree characteristics and other abiotic growth modulators.

The climatic determinants of larch deviated systematically from those of the other species. Both at Bosco/Gurin and Zermatt, the LMMs for larch included periods of significantly positive effects for previous year's August to October temperatures, with high absolute effects. Although the LMMs for spruce, Swiss stone pine and mountain pine also contained fall months, the period affecting growth occurred later, was shorter, and its absolute effects were smaller. This key difference between larch and the other species may be due to the deciduous nature of larch, relying much on stored carbohydrates to start the new growing season and producing a complete new set of needles (Kagawa, Sugimoto, & Maximov, 2006; King, Fonti et al., 2013; King, Gugerli et al., 2013). On the contrary, the other species retain their needles for several years. Random effects in relation to the fixed effects of previous late summer/fall temperature were high for all species and sites. Unlike previous correlation analyses, these findings suggest that the need for stored reserves may differ substantially among trees.

The amount of snow cover and the timing of snow melt are key factors determining growth processes at upper treeline (Hagedorn et al., 2014). The period of negative effects of spring temperatures on growth of larch at both sites, but also of spruce (although less distinct and with *p* values higher by several magnitudes) is likely due to a combination of temperature and the amount of snow during late winter/spring. Respiratory carbon losses were found to be directly linked to air temperature, as long as the root zone is too cold to permit the uptake of water (Wieser, 2012; Wieser & Bahn, 2004). Carbon balance analyses at treeline have shown losses during the late winter/spring for evergreen conifers but also for larches, which feature pronounced net carbon losses as late as June (Havranek, 1985; Wieser, 2012). This is especially the case because larches have no needles at the beginning of the growing season and therefore cannot counteract the carbon loss, unlike evergreen conifers (Wieser, 2012). The differences between larches at Bosco/Gurin and Zermatt presumably are a consequence of the sites' different precipitation regimes, with Bosco/Gurin having twice the annual precipitation sum and experiencing more extensive late winter snow than Zermatt (Blanchet, Marty, & Lehning, 2009). On the contrary, snow cover is believed to also have beneficial effects for tree growth under certain conditions. Although low temperatures in late spring shorten the growing season, the subsequent long‐lasting snow cover can provide greater water availability at the start of the growing season (Barbeito et al., 2012; Hättenschwiler & Smith, 1999). This hypothesis allows to explain the longer period of negative effects of spring temperature on tree growth at Hohgant compared to Bosco/Gurin. Trees at Hohgant grow on calcareous bedrock, which is heavily dominated by karst formations and lacks surface runoff. Therefore, they may depend more on water stored as snow than trees at Bosco/Gurin, which are situated on siliceous bedrock. In addition, deeper snow cover causes the trees to be better protected from freezing air temperatures. In a study on dwarf shrubs above treeline, low temperatures causing longer snow cover outweighed the positive effect of higher temperatures in spring that would result in a longer growing season (Wipf, Stoeckli, & Bebi, 2009). Low temperatures favoring snow cover further minimize the exposure of trees to wind abrasion, snow and ice damage, and winter desiccation (Holtmeier, 2009), and promote nutrient availability (Mack, Schuur, Bret‐Harte, Shaver, & Chapin, 2004; Sveinbjornsson, Hofgaard, & Lloyd, 2002). Another important factor controlled by spring temperatures is various forms of snow fungi (*Gremmeniella abietina*,* G. laricina*,* Phacidium infestans*, and *Herpotrichia juniperi*). They have been shown to strongly influence growth and mortality particularly of young trees (Barbeito et al., 2013), but possibly also to lead to a reduction of the photosynthetically active needle surface of larger trees, which may also lead to reduced growth rates.

Carbon allocation to growth versus reserves is a key strategic decision, especially for treeline trees (Felten, Hättenschwiler, Saurer, & Siegwolf, 2007; Landhausser, Pinno, Lieffers, & Chow, 2012). All tree species experienced conspicuously negative effects in late summer, paired with a high standard deviation of the corresponding random effects (Figure 3). The cambial activity of trees at treeline finishes around the end of July, however cell‐wall thickening and therefore the completion of the tree‐ring formation can last until mid‐October, even at treeline (e.g., Rossi, Deslauriers, Anfodillo, & Carraro, 2007). Although the share of the total ring width attributed to xylem differentiation is relatively smaller compared to cambial activity, it was found that up to 15% of the total ring width was produced during the cell‐wall thickening phase (Gruber, Zimmermann, Wieser, & Oberhuber, 2009). Larch at Bosco/Gurin stood out with a 3‐month period of negative effects and the highest absolute values across all sites. This phenomenon can be understood by examining the among‐tree variability (random effects). As mentioned above, the positive effects of previous fall temperatures reveal that some trees, most prominently larches, allocate a substantial amount of assimilates to reserves. The trees that deviated positively from the population mean in previous fall showed a negative deviation in the same months of the current year. This potential tradeoff applies to trees that allocate more assimilates to the carbohydrate reserves for the next year rather than to growth for the current year, provided that temperatures are favorable late in the growing season. The absolute values of the fixed effects emphasize again the high importance of reserve storage for the deciduous conifer larch compared to spruce, Swiss stone pine and mountain pine. Still, it has to be noted that the *p* values of the negative effects of current year's late summer are by several magnitudes higher than those of current and previous year's summer and still larger than those of current year's late winter/spring (Fig. S5).

### 4.3 Detection of long‐term growth trends

The growth rate trends at the uppermost part of the elevational gradients across all four treeline species were positive, but significant for larch at Bosco/Gurin and Zermatt only, and nearly significant for Swiss stone pine at Zermatt. Spruce and mountain pine did not yield significant trends in growth rates over time. Following the general hypothesis of temperature being the most important factor limiting tree growth at the treeline (e.g., Körner, 2012), as well as our findings regarding the relationship of temperature and growth (Figure 3) in conjunction with rising temperatures in months that affect tree growth (Figure 5), these trends are likely induced by improved growth conditions due to recent climate change. Although some studies suggest elevated atmospheric CO_2_ levels or increased nitrogen deposition to account for positive trends in tree growth rates of some species (e.g., Dawes et al., 2013), the timing of the growth increase at our study sites favors temperature as the key factor since the start of the positive trend (ca. 1950) does not coincide with a pronounced increase of CO_2_ (Bigler, 2016). Furthermore, the study sites are located in areas that experienced relatively small amounts of nitrogen deposition (Thimonier et al., 2010). Thus, our findings confirm the results of studies on the impact of climate change on tree growth in the Alps, attributing accelerated growth of Swiss stone pine (Motta & Nola, 2001; Vittoz, Rulence, Largey, & Freléchoux, 2008) and larch (Motta & Nola, 2001) to a rise in temperature.

Larch exhibits high climate sensitivity (Carrer & Urbinati, 2004), exceeding the one of Swiss stone pine, spruce, and mountain pine. This is well reflected in the temperature–growth LMMs (Figure 3) and may cause the species to profit more strongly from rising temperatures during the growing period (Figure 5) than the other species, and thus result in significant positive growth trends. On the contrary, Swiss stone pine, spruce, and mountain pine exhibited rather small effect sizes and therefore lower temperature sensitivity (Figure 3), leading to non‐significant growth reactions to the long‐term trend of increasing temperatures. However, these species still feature positive growth trends. Unlike the short‐term temperature–growth relationships, the long‐term development of growth over time showed relatively little among‐tree variability (Table 2). This may indicate that, regardless of the period affecting the growth of single trees, the general warming trend in all but 1 month (September) led to a low variability in growth trends over time and elevation. This hypothesis is confirmed by some larch trees, which strongly depend on reserve building that feature a weaker positive trend as mean September temperatures show a less pronounced increase (Figure 5) compared to other monthly mean temperatures.

Besides the temporal trends in ring widths, predictions based on the fixed effects also suggest overall smaller ring widths of trees growing at higher elevations (Figure 4). This decrease validates the use of elevation as a proxy in Equation (3) for varying temperature conditions experienced by the trees. Although this effect is significant for larch only, it reflects the temperature limitation of growth, which is valid for all trees (e.g., Körner, 2012) as temperature and length of the growing season decrease when approaching upper treeline. The small amount of among‐tree variability indicates a homogeneous pattern of decreasing ring widths with elevation across the populations at different sites.

In contrast to the similar growth trends across all four species at the upper limit of their range, they show a somewhat different trend at lower elevations, that is, further away from treeline. The predictions for larch showed decreasing growth trends, which may be due to the increasing stand density and thus higher competition (Figure 2). Improved growing conditions lead to higher stand density (Gehrig‐Fasel et al., 2007; Mazepa, 2005), which hampers growth of the early‐successional shade‐intolerant larch (Motta & Nola, 2001). In this context, the strengthening of the growth trend with decreasing elevation for spruce at Bosco/Gurin and Swiss stone pine at Zermatt can be related to the decrease in larch. Both Swiss stone pine and spruce are evergreen and relatively shade‐tolerant (Anfodillo et al., 1998), and they thus may substitute for larch at the lower end of the treeline ecotone. At the upper end of the treeline ecotone, the ability of larch to achieve high productivity throughout the growing season due to osmoregulation (Badalotti, Anfodillo, & Grace, 2000; Carrer & Urbinati, 2004) is advantageous with regard to its competitors. Although some studies suggest that larch may suffer from drought even at high elevations (Vittoz et al., 2008), it is unlikely to cause the diminishing positive growth trend with decreasing elevation observed in our study. Despite large differences in precipitation between the two sites (cf. Figure 1a,b,d), we found very similar patterns for larch growth at Bosco/Gurin and Zermatt (Table 2), suggesting that drought can be ruled out as a major cause for this interaction. The decline of larch is likely to be the first evidence of long‐suspected feedback effects due to climate change (cf. Wang et al., 2016). It must be noted, however, that also the cessation of human activity may result in forest densification (Gehrig‐Fasel et al., 2007) that negatively influences the performance of a shade‐intolerant species such as larch. A definite disentanglement of the causes for the stand densification is not possible with the data at hand. However, based on our rigorous approach to search for study sites, it is unlikely that changes in land use have caused an increase in stand density.

Detecting long‐term tree growth trends by means of dendroecological analyses poses a number of challenges, including biases that may be introduced by the sampling design (Bowman et al., 2013; Nehrbass‐Ahles et al., 2014) and the removal of biological growth trends (Bowman et al., 2013; Brienen et al., 2012; Peters et al., 2015). When sampling older trees in uneven‐aged stands like those at Bosco/Gurin, Hohgant and Zermatt, the trees' tradeoff between growth rate and longevity (c.f. Bigler & Veblen, 2009) tends to create a false‐positive growth trend due to the older trees in the sample being probably slow‐growing (“slow‐grower survivorship bias”) and young trees in the sample being mostly fast‐growing, as young slow‐growing trees are likely to be too small to be considered for sampling (“big tree‐selection bias”; Bowman et al., 2013; Brienen et al., 2012). We tackled these issues by first employing a stratified sampling design to obtain a “pseudo‐population” (Nehrbass‐Ahles et al., 2014), including selecting trees with DBHs as small as 5 cm, and second starting the trend analysis in the year 1850 only. Although Nehrbass‐Ahles et al. (2014) found a random sampling design to best address tree growth trends, in our case this would have led to a proportional representation and thus increased number of small and potentially young trees, which would carry limited and very similar information about the recent past. The “pseudo‐population” design was found to be also suited for growth trend analysis (Bowman et al., 2013; Nehrbass‐Ahles et al., 2014). Choosing the year 1850 as starting point arose from (i) the timespan being equal to that of the temperature–growth analyses, where this date was chosen due to the availability of climate data; (ii) ensuring a reasonable sample depth; and (iii) choosing a timeframe well below the known longevity across all tree species included in this study. Thus, these measures should limit the effects of both sampling biases.

For detrending the individual tree‐ring series, RCS was used, which is highly sensitive and reliable in detecting long‐term growth trends (Peters et al., 2015). Although RCS tends to dampen the slope of trends, the probability of false positives and negatives is small (Peters et al., 2015). In addition, the robustness of LMMs based on growth trend was investigated by ruling out predictive capabilities of supplementary site and tree parameters (cf. Section 2), that is, the long‐term growth trends contained in the ring‐width series were still best explained by time and elevation only.

In summary, we analyzed climatic treeline sites that do not currently show any strong evidence for an upward movement. The analysis of short‐term temperature–growth relationships using LMMs revealed a considerable amount of previously unquantified among‐tree variability, indicating differing strategies both between and within tree species related to the time when temperature actually affects ring‐width formation. Beyond this, the temperature effects on short‐term tree growth at natural treeline in three distinct regions of the Swiss Alps obtained from LMMs revealed a pattern that is consistent with findings from previous studies, regardless of the climate regime and tree species. In recent decades, positive growth trends across all study sites and species became evident, but they were significant for larch only, which is the most temperature‐sensitive species. The significant positive growth trend was most pronounced at the highest elevations of the study sites, which reflect the effects of ameliorated growth conditions. The observed declining growth trend with decreasing elevation that is most likely due to neighborhood interactions may indicate negative feedback effects of climate change on tree growth at these high‐elevation sites.

## CONFLICT OF INTEREST

None declared.

## AUTHOR'S CONTRIBUTIONS

Matthias Jochner: Design of the study, acquisition, analysis, and interpretation of data for the study; writing of the manuscript and revising it; final approval of the version to be published; agrees to be accountable for all aspects of the study.Harald Bugmann: Substantial contributions to the conception of the study, analysis, and interpretation of data; revising the manuscript and approving the final version to be published; agrees to be accountable for all aspects of the study.Magdalena Nötzli: Contributions to the acquisition and analysis of data; revising the manuscript and approving the final version to be published; agrees to be accountable for all aspects of the study. Christof Bigler: Design and conception of the study, acquisition, analysis, and interpretation of data for the study; revising the manuscript; final approval of the version to be published; agrees to be accountable for all aspects of the study.
