# Supplementary figures and images for: Among‐tree variability and feedback effects result in different growth responses to climate change at the upper treeline in the Swiss Alps
Source: Ecol Evol. 2017 Aug 30;7(19):7937–53. doi: 10.1002/ece3.3290 (PMC5632642; doi:10.1002/ece3.3290)

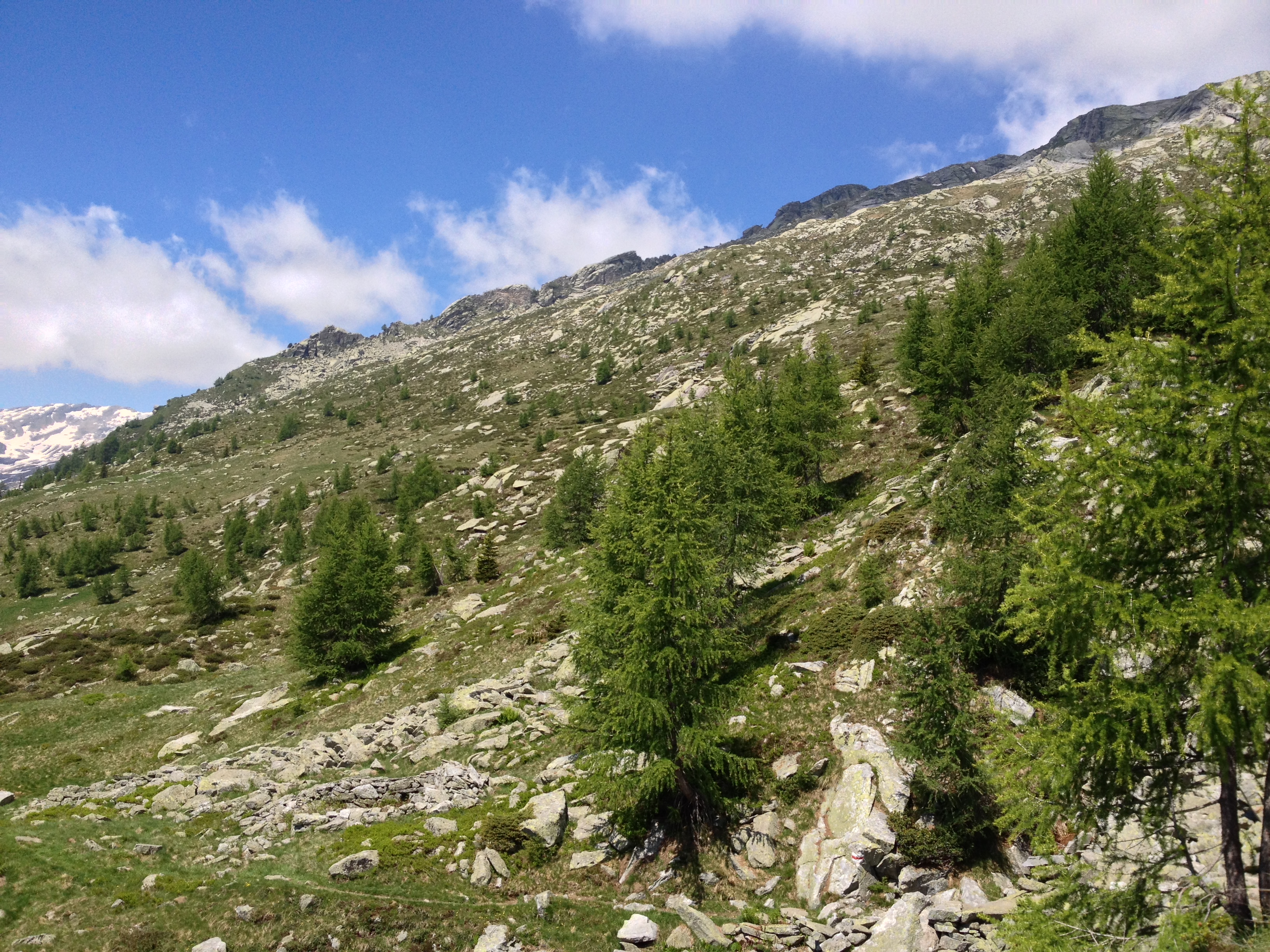

Supplement: Supplementary file 1 [file ECE3-7-7937-s001.JPG]

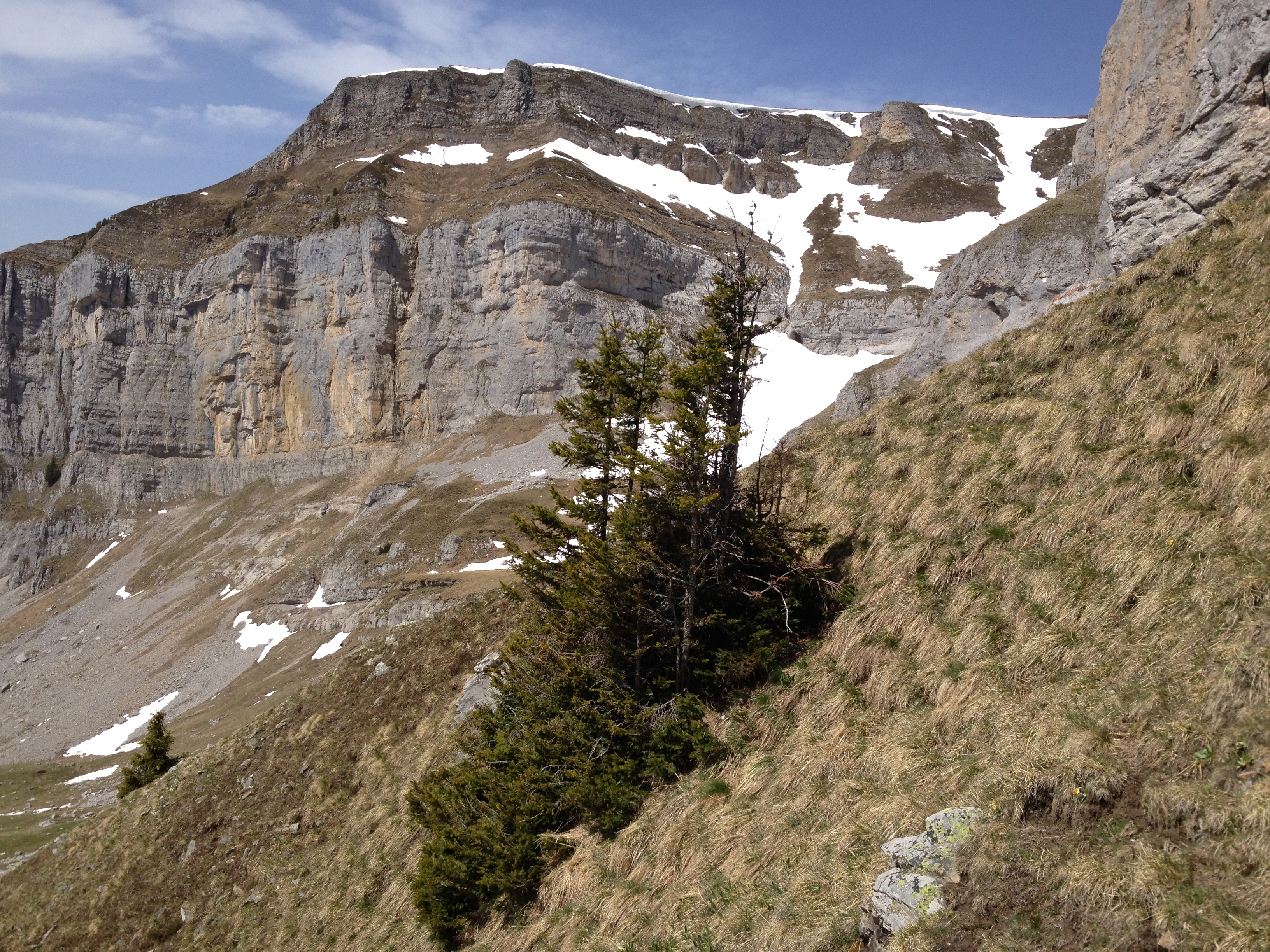

Supplement: Supplementary file 2 [file ECE3-7-7937-s002.JPG]

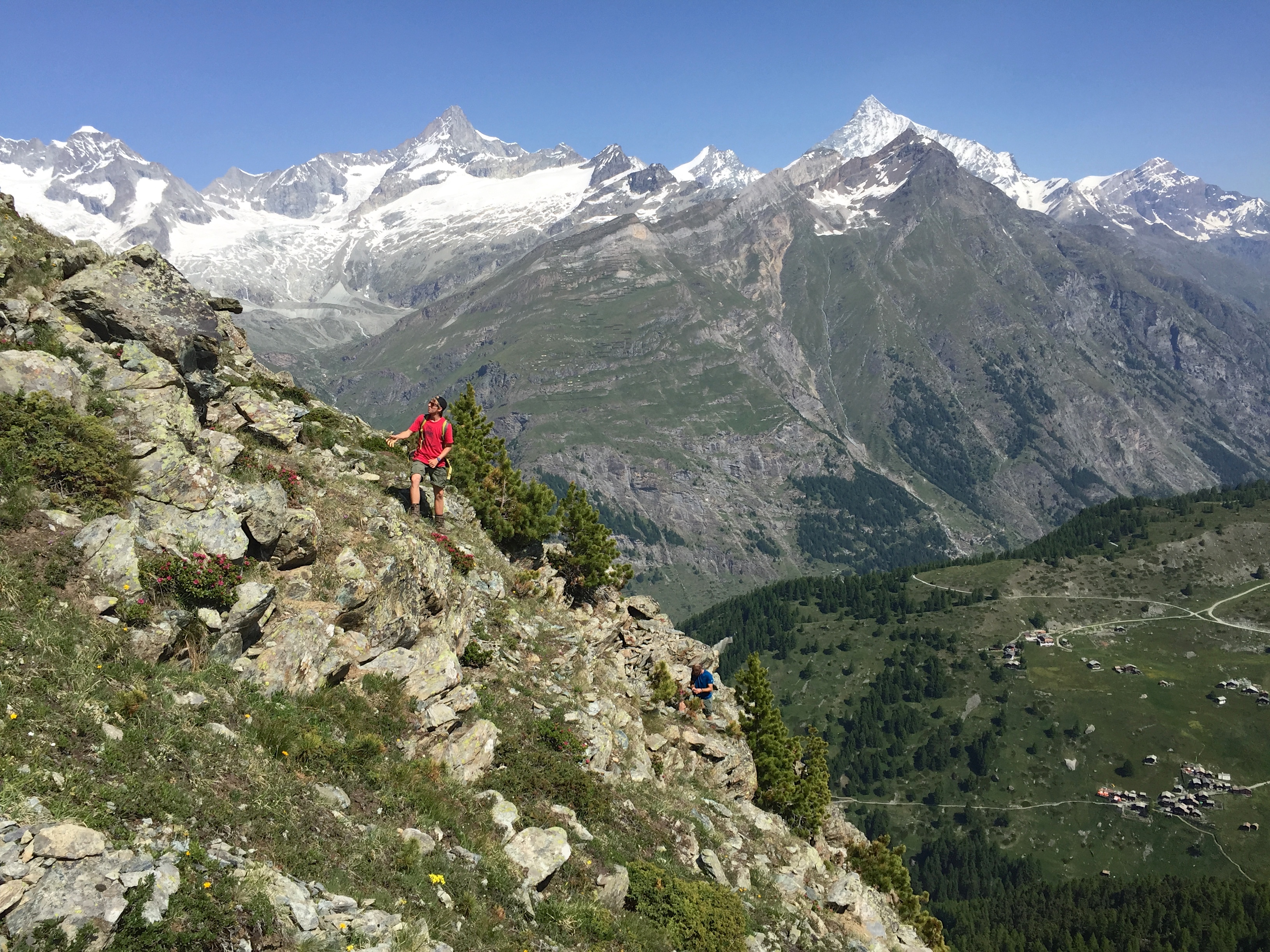

Supplement: Supplementary file 3 [file ECE3-7-7937-s003.JPG]

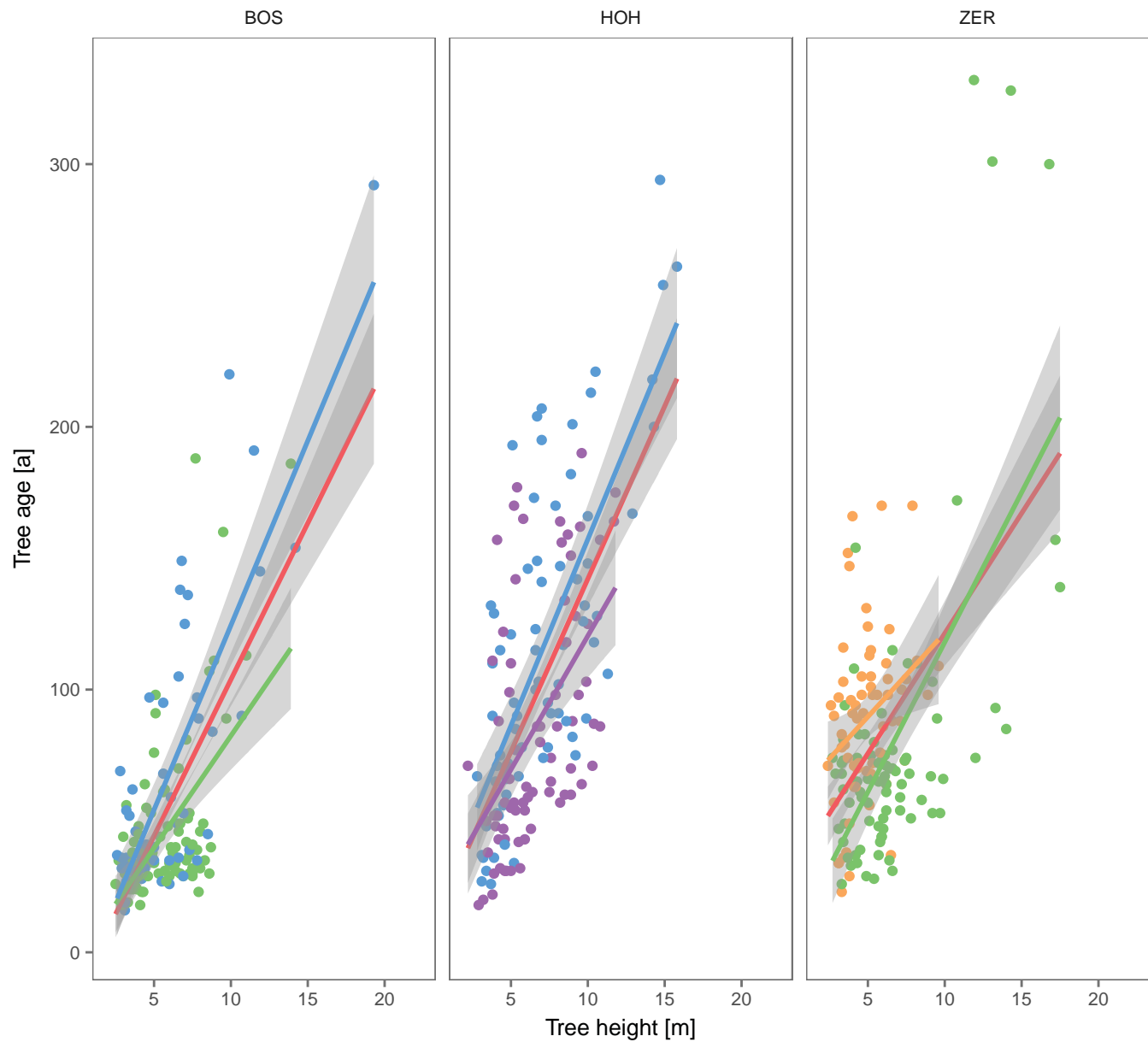

Tree species

Combined

*Larix decidua*

*Picea abies*

*Pinus cembra*

*Pinus mugo*

Supplement: Supplementary file 4 [file ECE3-7-7937-s004.pdf]

Larix decidua

Picea abies

Pinus  
cembraPinus  
mugo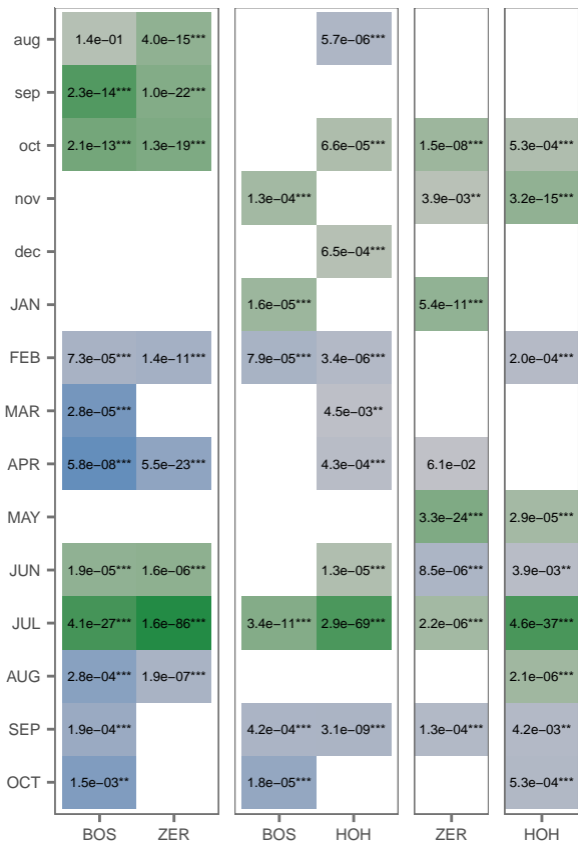

Fixed effect coefficient

-0.1

0.0

0.1

0.2

Supplement: Supplementary file 5 [file ECE3-7-7937-s005.pdf]
