# Supplementary material for: Among‐tree variability and feedback effects result in different growth responses to climate change at the upper treeline in the Swiss Alps
Source: Ecol Evol. 2017 Aug 30;7(19):7937–53. doi: 10.1002/ece3.3290 (PMC5632642; doi:10.1002/ece3.3290)
Supplement: Supplementary file 6 [file ECE3-7-7937-s006.docx]

Supporting information 4: Summary statistics of the tree-ring-width series. These include the number of trees of the species at each site, the mean series intercorrelation (± standard deviation; SD), the expressed population signal (EPS) and the mean series autocorrelation (± standard deviation).

| Study site | Species |  | Number of trees |  | Mean series intercorrelation ± SD |  | EPS* |  | Autocorrelation ± SD |
| --- | --- | --- | --- | --- | --- | --- | --- | --- | --- |
| Bosco/Gurin | Larix decidua |  | 95 |  | 0.52±0.2 |  | 0.9 |  | 0.45±0.2 |
|  | Picea abies |  | 60 |  | 0.44±0.18 |  | 0.87 |  | 0.68±0.21 |
| Hohgant | Picea abies |  | 85 |  | 0.46±0.16 |  | 0.91 |  | 0.76±0.14 |
|  | Pinus mugo |  | 83 |  | 0.43±0.15 |  | 0.93 |  | 0.61±0.24 |
| Zermatt | Larix decidua |  | 95 |  | 0.52±0.16 |  | 0.85 |  | 0.55±0.22 |
|  | Pinus cembra |  | 84 |  | 0.40±0.19 |  | 0.90 |  | 0.74±0.16 |

*Wigley *et al.* (1984) suggest an EPS value of 0.85 as an acceptable statistical quality
